# The regulation and function of the striated muscle activator of rho signaling (STARS) protein

**DOI:** 10.3389/fphys.2012.00469

**Published:** 2012-12-12

**Authors:** Marita A. Wallace, Séverine Lamon, Aaron P. Russell

**Affiliations:** Centre for Physical Activity and Nutrition Research, School of Exercise and Nutrition Sciences, Deakin UniversityBurwood, VIC, Australia

**Keywords:** ABRA, MS-1, sarcomere, exercise, disuse, cardiac muscle, skeletal muscle, smooth muscle

## Abstract

Healthy living throughout the lifespan requires continual growth and repair of cardiac, smooth, and skeletal muscle. To effectively maintain these processes muscle cells detect extracellular stress signals and efficiently transmit them to activate appropriate intracellular transcriptional programs. The striated muscle activator of Rho signaling (STARS) protein, also known as Myocyte Stress-1 (MS1) protein and Actin-binding Rho-activating protein (ABRA) is highly enriched in cardiac, skeletal, and smooth muscle. STARS binds actin, co-localizes to the sarcomere and is able to stabilize the actin cytoskeleton. By regulating actin polymerization, STARS also controls an intracellular signaling cascade that stimulates the serum response factor (SRF) transcriptional pathway; a pathway controlling genes involved in muscle cell proliferation, differentiation, and growth. Understanding the activation, transcriptional control and biological roles of STARS in cardiac, smooth, and skeletal muscle, will improve our understanding of physiological and pathophysiological muscle development and function.

## Identification of STARS

**Figure 1:**
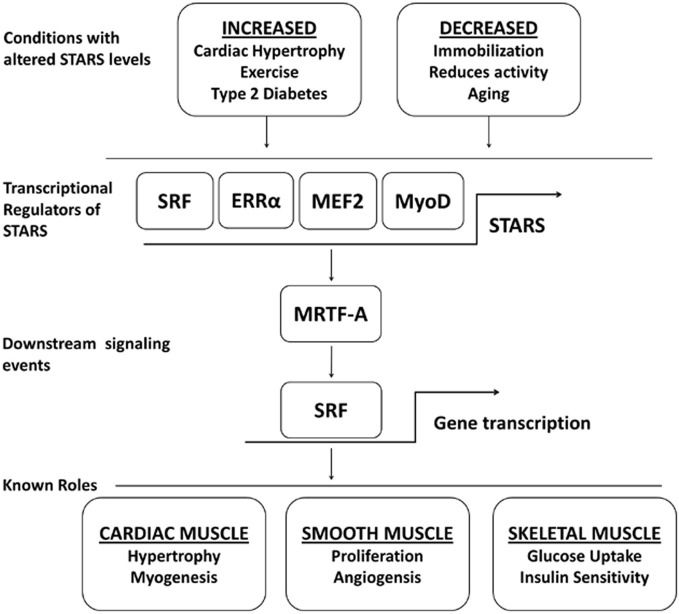
**A schematic outline highlighting the physiological and pathophysiological conditions that show a regulation of STARS, the transcription factors known to upregulate STARS, the downstream signaling events controlled by STARS, and the biological roles of STARS**.

The striated muscle activator of Rho signaling (STARS) protein, also known as Myocyte Stress-1 (MS1) and Actin-binding Rho-activating protein (ABRA), is a 43 kD protein, highly enriched in cardiac, skeletal, and smooth muscle (Arai et al., 2002; Mahadeva et al., 2002; Peng et al., 2008; Troidl et al., 2009). STARS was independently identified in 2002 using differential cDNA screening for novel genes expressed in the hearts of mouse embryos (Arai et al., 2002) and using molecular indexing to identify regulated genes following left ventricle (LV) pressure overload in the rat (Mahadeva et al., 2002). It is highly responsive to stress conditions and its ability to stimulate the serum response factor (SRF) (Arai et al., 2002; Kuwahara et al., 2005) transcriptional pathway makes this protein an interesting target for understanding physiological and pathophysiological muscle development. This review highlights our current understanding of STARS with a focus on its activation, transcriptional control, identifying physiological and pathophysiological conditions resulting in its regulation and the biological processes it influences. A schematic overview is shown in Figure 1.

## Localization and activation of STARS

In primary cardiomyocytes STARS locates to the sarcomere where it associates with the I-band, predominantly around the Z-disk and to a smaller extent in the M-line (Arai et al., 2002). When overexpressed, STARS binds actin in rat cardiomyocytes and non-muscle COS (monkey kidney cells) cells (Arai et al., 2002). Its function as an actin-binding protein has important consequences for intracellular signaling that may influence numerous cellular processes within muscle tissue. STARS, in conjunction with Rho, enhances actin polymerization by increasing the binding of G-actin to F-actin (Arai et al., 2002). The reduction in G-actin removes its inhibitory effect on the transcriptional co-activator, myocardin-related transcription factor-A (MRTFA), allowing its translocation to the nucleus (Arai et al., 2002; Kuwahara et al., 2005). Once in the nucleus, MRTF-A associates with SRF to promote transcription of its target genes (Kuwahara et al., 2005).

STARS protein activity is regulated by two actin-binding LIM (ABLIM) protein family members, ABLIM-2 and ABLIM-3 (Barrientos et al., 2007). Both ABLIM proteins co-precipitate with STARS in transfected COS cells, strongly bind to F-actin in C2C12 cells and localize to the sarcomere in mouse skeletal muscle (Barrientos et al., 2007). The ABLIM proteins can also enhance STARS-dependent SRF-activation. It has been suggested that the ABLIM proteins regulate STARS activity by assisting its binding to the sarcomere (Barrientos et al., 2007). As the sarcomere plays a critical role in sensing biomechanical stress and activating signaling pathways in skeletal muscle, STARS could possibly act as a link between contractile function and intracellular signaling in muscle cells.

The actin binding of STARS requires two separate but co-dependent regions in the C-terminal end of the protein, located between the amino acid sequences 234–279 and 346–375 (Arai et al., 2002). These regions contain two independent F-actin binding domains, actin binding domain 1 and 2 (ABD1/ABD2) (Fogl et al., 2011). ABD1 (fragment 193–296) binds with higher affinity to F-actin when compared to ABD2. ABD1 does not adopt a well-folded structure until it is bound to F-actin (Fogl et al., 2011), while ABD2 (fragment 294–375), the most C-terminal fragment, is independently folded (Fogl et al., 2011). It has been hypothesized that ABD1 could completely fulfill the actin-binding function of STARS in muscle, therefore, leaving ABD2 available for other potential functions within muscle (Fogl et al., 2011). While STARS is predominantly cytosolic, we (Wallace et al., 2011) and others (Arai et al., 2002) have observed its location in the nucleus. It is possible that the nuclear role or function of STARS is controlled by ABD2, however, this remains to be determined. The functional significance of STARS within the nucleus is currently unclear but leaves the possibility that STARS itself may act as a transcriptional co-activator or transcription factor. Future studies are required to determine if STARS can bind DNA.

## Transcriptional regulation of STARS

**Figure 2:**
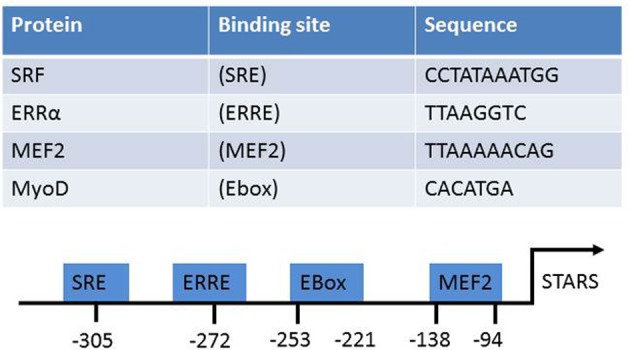
**The known transcriptional activators of STARS, their target sequences, and location on the STARS promoter**.

The STARS promoter contains binding sites for several muscle-enriched transcription factors (Figure 2). Gene expression of STARS is diminished in the heart of *Mef2c* null mouse embryos. This suggests that *Mef2c* may be involved in the transcriptional regulation of STARS gene expression, at least in cardiac muscle (Kuwahara et al., 2007). Further analysis confirmed the existence of two regions that synergistically mediate MEF2C activation of STARS transcription (Kuwahara et al., 2007). Two conserved E-boxes have also been identified within the proximal 1.5 kbp of the 5′ upstream STARS promoter sequence (Ounzain et al., 2008). These two sites are required for recruiting MyoD and subsequently activating the STARS promoter during myogenic differentiation in C2C12 muscle cells. Recent work from our group identified a putative estrogen-related response element (ERRE) binding site on the STARS promoter (Wallace et al., 2011). Over expression of constitutively active estrogen-related receptor-α (ERRα) in C2C12 myotubes increased STARS mRNA levels. Over expressing peroxisome proliferator-activated receptor-γ coactivator-1α (PGC-1α), with and without the shRNA knock-down of ERRα in C2C12 myotubes, demonstrated that STARS is under the control of a PGC-1α/ERRα transcriptional program. STARS can also regulate its own transcription via a feed-forward mechanism that requires SRF binding to the serum response element (SRE) on the STARS promoter (Chong et al., 2012). Conversely, STARS is repressed in embryonic, neonatal, and adult hearts by GATA4 (Ounzain et al., 2012). Depletion of GATA4 allows the pathological up regulation of STARS (Ounzain et al., 2012).

## Physiological and pathological conditions that influence STARS expression

### Cardiac muscle hypertrophy

Cardiac hypertrophy is an adaptation of the heart in response to an increased workload, stress, or injury. This adaptation is initially beneficial but sustained cardiac hypertrophy eventually results in impaired cardiac function, partially due to cell death caused by apoptosis (Dorn, 2009). STARS is increased in several models of cardiac hypertrophy and myopathy. Left ventricle hypertrophy (LVH) in rats, stimulated by pressure overload by aortic banding, caused a pronounced early up-regulation of STARS within 1 h and a considerable 3-fold increase in STARS at 4 h post banding (Mahadeva et al., 2002). This elevation of STARS mRNA had returned to control levels before 24 h post banding. The transient increase in STARS gene expression was observed prior to any detectable increase in LV mass which did not occur until at least 4 days post banding. This suggests that STARS may detect the initial mechanical stress and its activation would signal to mechanisms that directly cause the cardiac remodeling response to LVH. Transgenic (Tg) mice expressing constitutively active calcineurin, under the control of a cardiac-specific α-myosin heavy chain (α-MHC) promoter (Cn-Tg mice), also develop cardiac hypertrophy and cardiomyopathy (Kuwahara et al., 2007). In this model STARS is also 8–9-fold higher in the hearts of Cn-Tg mice when compared to wild type littermates (Kuwahara et al., 2007). However, this cardiac hypertrophy only occurs when the mice are subjected to pressure overload. A similar observation has been made in humans with STARS mRNA significantly higher in human hearts with idiopathic cardiomyopathy when compared to normal control hearts (Kuwahara et al., 2007). *In vitro* it has been shown that overexpressing STARS increases the size of H2c9 rat myocardial cells (Koekemoer et al., 2009). While the mechanism behind this cardiomyocyte hypertrophy was not established, STARS overexpression did not affect proliferation, but it did reduce chemically induced apoptosis (Koekemoer et al., 2009). STARS may also regulate muscle cell hypertrophy by influencing protein synthesis and degradation, however, this has not been experimentally determined. These observations suggest that STARS is an early marker of cardiac remodeling in response to increased mechanical stress. The fact that endogenous STARS is rapidly and transiently increased in response to external stress signals suggests that it may have a protective and a physiological adaptive role in muscle cells. However, forced and uncontrolled up-regulation of STARS may result in maladaptation.

### Exercise, disuse, and aging

The adaptation of skeletal muscle to changes in mechanical stress, such as muscle contraction or immobilization, requires the sensing and the transduction of the extracellular stress signals into the cell in order to generate the appropriate physiological response. Our group first observed that STARS mRNA levels significantly increased in the vastus lateralis muscle following 8 weeks of hypertrophy-inducing resistance exercise training in humans (Lamon et al., 2009). Similarly skeletal muscle STARS gene expression is increased (Pollanen et al., 2010) in post-menauposal women following 12 months of hypertrophy-inducing plyometric power training (Sipila et al., 2001). Whether STARS directly causes skeletal muscle hypertrophy has not been established. With respect to acute single-bout exercise, STARS gene expression increases 10-fold when measured 3 h after eccentric exercise (MacNeil et al., 2010). Work from our group has also shown that STARS mRNA and protein levels are increased 3 h following an acute bout of single-leg endurance exercise; both returning to basal levels 24 h post-exercise (Wallace et al., 2011). This transient increase in STARS following contraction-induced mechanical stress may provide a protective mechanism to reduce the risk of contractile damage to the sarcomere or be required to activate intracellular signals responsible for muscle adaptation to exercise.

Limb immobilization drastically reduces the exposure of skeletal muscle to mechanical stress factors. Hind limb suspension decreased STARS mRNA expression in rat soleus muscle within the first 24 h and remains elevated for 48 h. However, a reduction in muscle weight was not observed until 48 h post intervention (Giger et al., 2009). Whether the decrease in STARS is a cause or a consequence of muscle wasting is yet to be established. In contrast, 20 days of hind limb suspension in humans, resulting in a 10% loss in muscle mass, did not reduce STARS mRNA levels (Sakuma et al., 2009). We have observed that reducing mechanical stress due to cessation of exercise training for 8 weeks results in a loss of muscle mass and a reduction in STARS mRNA (Lamon et al., 2009).

STARS is reduced in skeletal muscle from of aged mice (Sakuma et al., 2008) and pigs (Peng et al., 2008). As STARS binds and stabilizes actin and the sarcomere, it has been suggested that it protects against contraction-induced damage (Arai et al., 2002). Muscle from older animals is more susceptible to contraction-induced damage, partially due to a mechanically compromised sarcomeric structure that is less able to withstand stretch (Lynch et al., 2008). Older muscle also repairs less efficiently which is attributed to its reduced capacity to promote satellite cell activation, proliferation, and differentiation, thereby contributing to its impaired regenerative capacity and reduced muscle mass (Grounds, 1998; Welle, 2002). The reduction in STARS in older muscle may be a factor contributing to the increased susceptibility of contraction-induced muscle damage as well as its attenuated muscle cell proliferation and repair.

### Insulin resistance

STARS gene expression is upregulated in skeletal muscle of patients with type 2 diabetes when compared with healthy family members (Jin et al., 2011). In rodents, STARS expression is also increased in mice made insulin resistant by high-fat feeding (Jin et al., 2011) as well as in the heart of streptozotocin (STZ)-induced type I diabetic mice and in db/db type II diabetic mice (Ounzain et al., 2012). Knock-down of STARS in L6 myotubes enhances insulin signaling, as measured by increased insulin stimulated Akt phosphorylation as well as basal and insulin-stimulated glucose uptake (Jin et al., 2011). Additionally, the reduction in STARS increases plasma membrane GLUT4 localization in basal and insulin stimulated conditions. Presently, it is difficult to determine if the increase in STARS in diabetic muscle is a consequence of or directly contributes to insulin resistance.

## Other biological roles of STARS

### Angiogenesis

Fluid shear stress (FSS)-induced collateral growth during arteriogenesis is associated with an increase in STARS gene expression (Troidl et al., 2009). This induction of STARS is abolished with the nitric oxide (NO) inhibitor L-NAME, suggesting that STARS transcription may be controlled by NO. The local intracollateral over expression of STARS ameliorates collateral conductance in rabbits following femoral artery ligature. In contrast, mice with an ablation of STARS have impaired arteriogenesis (Troidl et al., 2009). These observations extend the role of STARS to arterial structure and function.

### Smooth muscle cell proliferation

Overexpression of STARS in porcine smooth muscle cells (SMCs) increases proliferation (Troidl et al., 2009). The ability of STARS to enhance SMC proliferation was also observed in the A10 rat vascular SMC line, but not in porcine aortic endothelial cells (Troidl et al., 2009) or in the H9c2 rat cardiac cell line (Koekemoer et al., 2009). At present the effect of STARS on skeletal muscle cell proliferation has not been investigated. The potential of STARS to promote muscle cell proliferation requires further validation *in vivo*. Furthermore, identification of the downstream molecules activated by STARS that regulate proliferation is required. STARS may play an important role in sensing mechanical stress and up-regulating important pathways controlling muscle growth and regeneration. It also appears that the biological role/s of STARS may depend on the muscle cell type investigated.

## Conclusion

Continual adaptations such as growth, repair, and maintenance of cardiac, smooth, and skeletal muscle are required throughout the lifespan for the maintenance of healthy living and longevity. For healthy adaptation to occur the muscle cells must be able to detect the extracellular stress signals and efficiently transmit them to appropriate intracellular transcriptional programs. The STARS protein appears to be a key player in the detection and transmission of adaptive stress signals in muscle cells. However, forced chronic overexpression of STARS appears to make the muscle cells hypersensitive to adaptive external stress and may result in a maladaptive response. These observations suggest that if STARS was to be considered a therapeutic target to enhance muscle growth and repair then it would require a precise and conditional method of induction or suppression.

### Conflict of interest statement

The authors declare that the research was conducted in the absence of any commercial or financial relationships that could be construed as a potential conflict of interest.
